# Bread Consumption Is Associated with Elevated Blood Pressure among Adults Living in Mexico City–A Sub-Analysis of the Tlalpan 2020 Study

**DOI:** 10.3390/nu10121969

**Published:** 2018-12-13

**Authors:** Xochitl Ponce-Martínez, Eloisa Colin-Ramirez, Paulina Sánchez-Puerto, Susana Rivera-Mancía, Raúl Cartas-Rosado, Oscar Infante-Vázquez, Maite Vallejo-Allende, Jesús Vargas-Barrón

**Affiliations:** 1 Sociomedical Research Department, National Institute of Cardiology Ignacio Chávez, Mexico City 14080, Mexico; xochitl.ponce@gmail.com (X.P.-M.); ppuerto22@hotmail.com (P.S.-P.); syriverama@conacyt.mx (S.R.-M.); maite_vallejo@yahoo.com.mx (M.V.-A.); 2 Cátedras CONACYT, National Council of Science and Technology, Mexico City 03940, Mexico; 3 Electromechanical Instrumentation Department, National Institute of Cardiology Ignacio Chávez, Mexico City 14080, Mexico; rcartas@gmail.com (R.C.-R.); osinfa@yahoo.com (O.I.-V.); 4 Division of Research, National Institute of Cardiology Ignacio Chávez, Mexico City 14080, Mexico; ecovarjes@gmail.com

**Keywords:** bread consumption, sodium intake, elevated blood pressure, Mexican population

## Abstract

Excessive dietary sodium is associated with elevated blood pressure (EBP). Bread products are identified as one of the main sources of daily sodium intake. The objective of this cross-sectional study was to evaluate the association between bread and others cereal products consumption with EBP. Frequency intake of a standard serving of bread and other cereal products was recorded and categorized as: ≤3 times/month or never (reference category group) and ≥ once/week. EBP was defined as systolic blood pressure (SBP) ≥120 mmHg and/or diastolic blood pressure (DBP) ≥80 mmHg. Raw and adjusted odds ratios (OR) for the association between consumption of the studied food products and blood pressure status were estimated. Overall, 2011 participants aged 37.3 ± 9.1 years old were included. In the models adjusted for relevant covariates, consumption of one piece of bolillo or telera (OR = 1.39; 95% CI = 1.01–1.89) ≥ once/week was associated with an increased risk of EBP, compared to the reference category. Also, participants consuming one bowl of high-fiber breakfast cereal once/week were less likely to have EBP (OR = 0.73; 95% CI = 0.53–0.98). Initiatives to reduce sodium levels in bread products such as bolillo and telera are needed in Mexico to help manage the cardiovascular risk at the population level.

## 1. Introduction

Hypertension (HTN) is a major risk factor for the development of cardiovascular, cerebrovascular, and kidney diseases [1]. Globally, it is estimated that 89.9 million years of disability among women and 122.2 million among men were attributable to high systolic blood pressure in 2016 [2]. Worldwide HTN prevalence among subjects >20 years old was 31.1% in 2010; a decrease of 2.6% in HTN prevalence was reported in high-income countries, whereas an increase of 7.7% was observed in low- and middle-income countries between 2000 and 2010 [3]. In Mexico, the prevalence of HTN has increased over recent years from 30.1% in 2000 to 31.6% in 2006 and 31.4% in 2012 [4].

There is a large body of evidence linking excessive sodium intake to elevated blood pressure (EBP) and cardiovascular events [5,6]. Importantly, a high–sodium diet accounted for 4.2% of global deaths in 2016 [2]. In Mexico, in an adult population, dietary sodium intake has been estimated at 3150 mg/day (3734.9 mg/day in men and 2875.3 mg/day in women), as measured by 24 h urinary sodium excretion [7]. In this regard, recent data reported that bread (savory bread and sweet bakery goods) was the major contributor to daily sodium intake among an adult Mexican population, accounting for 16% of the total sodium intake, followed by processed meat (8%) and natural cheeses (5%) [8]. These results were similar to those observed in diverse western populations [9,10,11,12,13], where bread [10,11,12,13] and bread and cereal-derived products combined [9] were found to be the major contributors for daily sodium intake, although the percentage of contribution varies across these countries. The impact of bread consumption on daily sodium intake is probably not only due to the salt added for preservation and sensory purposes [14], but also due to its large consumption. Indeed, in 2012, per capita white bread consumption (packaged sliced bread and bolillo/telera) in Mexico was reported to be 24.4 kg/year, with a mean sodium content in this food product of 520 mg/100 g [15]. Sodium content in these food products has not been documented since then.

Despite the recognition of bread and cereal products as a relevant food source for sodium intake [14,16,17,18], there is still little information available about their effects on blood pressure. Most existing studies have focused on the effects of whole and refined grains on metabolic indicators and cardiovascular risk [19,20], but not bread in particular, which is largely consumed in the western diet. Evidence of the impact of bread consumption on blood pressure will serve to support the implementation and proper monitoring of existing initiatives to reduce sodium content in bread and other cereal products [15,21]. The underlying hypothesis of this study is that a higher frequency of intake of bread and other cereal-derived products is associated with a higher risk of elevated blood pressure (EBP). The objective of this study was to estimate the association between frequency of intake of bread and other cereal products with elevated blood pressure in an adult population in Mexico City.

## 2. Materials and Methods

### 2.1. Subjects and Study Design

This cross-sectional analysis was based on baseline data from participants enrolled in the Tlalpan 2020 cohort, which is an ongoing observational, prospective, longitudinal study of risk factors for hypertension incidence in a population of adults aged 20–50 years old living in Mexico City. This study is being conducted by the Instituto Nacional de Cardiología Ignacio Chávez (INCICh) [*National Institute of Cardiology Ignacio Chávez*]; enrolment started in September 2014. Subjects with systolic blood pressure (SBP) ≥ 140 mm Hg and/or diastolic blood pressure (DBP) ≥ 90 mm Hg, diabetes mellitus, dysthyroidism, cerebrovascular disease, ischemic cardiomyopathy, acute coronary syndrome, active cancer with an effect on survival, cognitive or mental disability, pregnant women, and those taking medications that modify blood pressure, or with any other condition which, in the opinion of the researchers, could preclude compliance with the protocol, were excluded from the cohort. Methodology details of the Tlalpan 2020 cohort were published elsewhere [22]. For the purpose of this cross-sectional analysis, participants recruited in the cohort between September 2014 and June 2017 were included. Also, individuals who were screened but excluded from the Tlalpan 2020 cohort due to hypertension (SBP ≥ 140 mm Hg and/or DBP ≥ 90 mm Hg) were included in the present analysis in order to capture a wider range of blood pressure levels among participants. Individuals with incomplete dietary data were excluded from this sub-analysis. All participants gave their written informed consent to be recruited in the cohort. The study was conducted in accordance with the Declaration of Helsinki, and the protocol was approved by the Institutional Bioethics Committee of the INCICh (REF. 13-802).

Clinical assessments and collection of biologic samples and all data related to this study are conducted at the INCICh.

### 2.2. Blood Pressure Assessment and High Blood Pressure Definition

Blood pressure was measured three times in the left arm with a calibrated mercury sphygmomanometer, with a 3-min interval between each measurement, with the subject remaining seated for at least 10 min prior to the first evaluation. The averages of the three SBP and DBP measurements were calculated and participants were classified as having normal (SBP < 120 mmHg and DBP < 80 mmHg) or elevated blood pressure (EBP) (SBP ≥ 120 mmHg and/or DBP ≥ 80 mmHg) [23]. 

### 2.3. Anthropometric Data 

Weight, height, and waist circumference (WC) were assessed by trained personnel with subjects fasting, shoeless, and wearing a hospital gown, according to the procedures described by ‘The International Society for the Advancement of Kinanthropometry [24,25]. Weight was measured with a mechanical column scale (SECA 700) with a capacity of 220 kg and a precision of 0.05 kg. A stadiometer SECA 220 was used to assess height in meters and the measurement was recorded to the nearest millimeter. WC was measured with a measuring tape made of glass fiber BodyFlex, with length of 150 cm and precision of 1 mm. Body mass index (BMI) was computed by dividing total body weight (kilograms) by height squared (square meters).

### 2.4. Dietary Information

Dietary intake was evaluated using a semi-quantitative food frequency questionnaire (FFQ) designed and validated for the Mexican population [26], which was administered by trained personnel. The FFQ includes 116 foods and beverages classified into ten different categories: 1. Dairy products; 2. Fruits; 3. Eggs, meat, fish, poultry, and processed meat; 4. Vegetables; 5. Legumes; 6. Cereal products; 7. Sweets and candies; 8. Beverages; 9. Oils; and 10. Mexican foods. This FFQ evaluates ten frequencies of consumption: never, less than once per month, 1–3 times per month, once per week, 2–4 times per week, 5–6 times per week, once per day, 2–3 times per day, 4–5 times per day, and 6 or more times per day, of a standardized portion of each food or beverage (e.g., 1 cup of milk, 1 piece of orange, 1 piece of egg, etc.) during the year prior to the interview. Data collected in the FFQ were processed using the nutrient software program ‘Sistema de Evaluación de Hábitos Nutricionales y Consumo de Nutrimentos’ (SNUT) (Evaluation of Nutritional Habits and Nutrient Consumption System), developed by the National Institute of Public Health of Mexico [27], to estimate daily energy (kcal), macronutrient (grams of protein, carbohydrates, and total fat) and micronutrient (mg of sodium, potassium and magnesium) intake. 

### 2.5. Frequency of Intake of Bread and Cereal Products Definition

The ‘cereal products’ food category in the FFQ includes: 1 piece of corn tortilla, 1 piece of wheat flour tortilla, 1 slice of packaged white bread, 1 slice of packaged whole-grain bread, 1 piece of bolillo or telera (a type of savory bread traditionally made in Mexico, similar to baguette), 1 piece of sweet bakery goods, 1 bowl of oats (plain and flavored), 1 bowl of refined-grain breakfast cereal, and 1 bowl of high-fiber breakfast cereal (including granola). For the purpose of this analysis, frequency of consumption of each of these food items was re-categorized into four categories as follows: (1) ≤3 times per month or never; (2) 1–4 times per week; (3) 5–6 times per week; and (4) ≥ once per day. Additionally, it was further re-categorized as: (1) ≤3 times per month or never and (2) ≥once per week.

### 2.6. Biochemical Evaluation

Fasting serum levels of total (TC), high density lipoprotein (HDL) and low density lipoprotein (LDL) cholesterol, triglycerides, and glucose were determined for each participant after a 12-h fasting period, using automated analyzers in the Central Laboratory of the INCICh. 

### 2.7. 24 h Sodium Urinary Excretion

For descriptive purposes, we collected data on 24 h urinary sodium excretion. Participants were asked to collect a 24 h urine sample the day prior to their baseline study visit at the INCICh. Detailed information about the correct form of collecting the urine sample was provided to the participants (discard the first urine in the morning and collect all urine for a period of 24 h, including the first void of the baseline visit). Participants received a preservative free container and were asked to store the collected urine in a cool place during the collection period. Urinary sodium was determined using the ion selective electrode method, urinary creatinine was measured by Jaffe’s colorimetric assay in automated analyzers. Urine samples were considered as complete when urinary creatinine levels were within the standard creatinine excretion rate for sex (15–25 mg/kg/24 h and 10–20 mg/kg/24 h for women) [28]. 

### 2.8. Other Variables 

Other variables such as education level and alcohol and tobacco use were also collected during face-to-face interview conducted by trained research personnel. Education level was documented as the last study degree achieved. Current alcohol consumers were all participants who, at the time of the interview, reported to currently consume alcohol regardless of the frequency of intake (daily, every other day, every weekend, every two weeks, once a month, or less than once a month). Current cigarette smokers were those persons who reported to have smoked at least 100 cigarettes in their lifetime and that, at the time of interview, smoked either every day or some days [29]. 

### 2.9. Statistical Analysis

Descriptive statistics of the study sample are presented as means ± standard deviation for continuous variables, and proportions for categorical variables. Comparisons between men and women were performed by using the independent sample t-test for continuous variables and chi-square test for categorical variables.

A one-way analysis of variance was performed to test the differences in mean daily dietary sodium intake (by FFQ) between the four categories of frequency of intake of the studied food products. Multiple comparisons between categories were conducted by the Bonferroni method. Additionally, univariable and multivariable linear regression analyses were performed to test the linear association between the categories of frequency of intake of each studied food product and daily dietary sodium intake (mg). Regression coefficients (β) and 95% confidence intervals (95% CI) for β were estimated for all food frequencies, considering the frequency of intake ‘≤3 times/month or never’ as the reference category. All multivariable linear models were adjusted for age, sex, BMI, daily energy intake (kcal/day), and frequency of consumption of bacon, ham, sausages and cheeses (as measured in their original 10-option frequency scale). Since only one 24 h urine sample was collected from each participant, and frequency of intake of the studied food products was evaluated by the FFQ for a 1-year timeframe, daily dietary sodium intake estimates considered for these analyses were based on the assessment by the FFQ instead of the 24-h urinary sodium excretion.

Univariable and multivariable logistic regression analyses were conducted to estimate the unadjusted and adjusted odds ratios (OR) and 95% CI of having EBP (SBP ≥ 120 mmHg and/or DBP ≥ 80 mmHg) among participants that reported to consume bread and other cereal-derived products of interest at least once per week, as compared to those who reported to eat these foods ≤3 times per month or never. Multivariable models were adjusted by factors known to be associated with blood pressure such as age, sex, education level, BMI, alcohol consumption, and tobacco use, as well as relevant dietary factors such as intake of energy (kcal/day), potassium (mg/day) and magnesium (mg/day). Also, we adjusted for frequency of intake of bacon, ham, sausages, and cheeses (as measured in their original 10-option frequency scale), since processed meat and cheeses were reported to be among the top 3 dietary sources of sodium in an adult population of Mexico City, as previously mentioned in the introduction section [8]. A *p* value below 0.05 was considered as statistically significant. All statistical analyses and data processing were performed using STATA, version 14 (Stata Corp., College Station, TX, USA) for Mac. 

## 3. Results

### 3.1. Participant Characteristics

**Figure 1:**
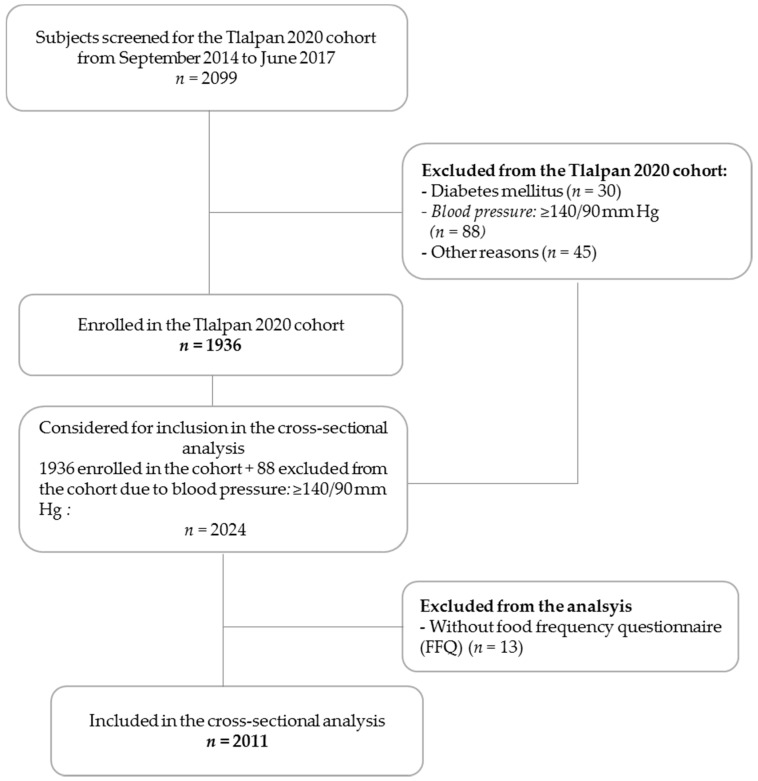
Participants flow chart.

We included in this analysis a total of 2011 participant (Figure 1), of which 1274 (63.4%) were women. 

Demographic and clinical characteristics for the overall study sample and stratified by sex are shown in Table 1. Overall, the mean age was 37.3 ± 9.1 years old, most of the participants (65.4%) were either overweight or obese, and the prevalence of EBP was 26.7%. Comparison between sexes showed that men were younger and reported higher frequencies of alcohol intake, tobacco use, overweightness and obesity, overall EBP, SBP ≥ 120 mm Hg, and DBP ≥ 80 mm Hg. Additionally, we observed greater mean values of systolic and diastolic blood pressure, waist circumference, fasting plasma glucose, triglycerides, total cholesterol, and LDL-c, as well as a lower mean HDL-c among men than women. Table 2 shows key dietary intake data stratified by sex. Men exhibited a higher mean daily intake of energy, macronutrients, and micronutrients, including dietary sodium, while women reported to eat bacon, sausages, ham, and chesses (except for fresh cheese) less frequently. Of all 2011 participants, 1492 (74.2%) provided a complete 24 h urine sample as determined by sex-specific creatinine excretion rate; mean urinary sodium excretion was 3116.3 ± 1273.3 mg/day, and it was higher among men than women.

### 3.2. Association between Frequency of Intake of Bread and Other Cereal Products and Daily Dietary Sodium Intake

The analysis of variance showed that mean daily dietary sodium intake (as measured by the FFQ) was different among categories of frequency of intake for all food products. When the linear association between frequency of intake of the studied food products and daily sodium intake was tested, considering a frequency of intake ≤ 3 times per month or never as the reference category, all unadjusted regression coefficients (β) for daily sodium intake (mg/day) increase as the category of frequency of intake of each food product does so as well, except for corn tortilla, oats, refined-grain breakfast cereal and high-fiber breakfast cereal. When all models were adjusted for key variables, only the frequency of intake of packaged whole-grain bread and oats showed a positive linear association with daily dietary sodium intake. Importantly, frequency of intake of packaged white bread no longer exhibited a linear association with sodium intake after adjustment; however, the β for the category representing the highest frequency of intake (≥once per day) remained positive and significant for this food item. In the case of bolillo or telera, the adjusted β increases as the frequency of intake does so, but only category 4 (≥ once per day) was statistically significant. High-fiber breakfast cereal did not show a linear association with daily sodium intake either, although it is important to highlight that category 4 (≥ once per day) showed a positive and meaningful β for daily sodium intake. Regression coefficients for all food items are shown in Table 3.

### 3.3. Association between Frequency of Intake of Bread and Other Cereal Products and EBP

Table 4 shows the unadjusted and multivariable-adjusted ORs (95% CI) for overall EBP among participants consuming bread and other cereal-derived products at least once per week. In the univariable model, subjects consuming 1 piece of bolillo or telera or 1 piece of sweet bakery goods ≥once per week had a significantly increased risk of having EBP, as compared to those consuming these food items ≤3 times per month or never. In contrast, consumption of 1 bowl of high-fiber breakfast cereal ≥once per week was associated with a significant decreased risk of having EBP when compared to the reference category (≤3 times per month or never). After adjustment for key covariates, the association of EBP with the consumption of 1 piece of bolillo or telera or 1 bowl of high-fiber breakfast cereal ≥once per week remained significant; however, the association between EBP and the consumption of sweet bakery goods was attenuated.

## 4. Discussion

In this study of an adult population from Mexico City, we found a 1.39-fold increased risk of EBP associated with the consumption of 1 piece of bolillo or telera at least once per week, as compared to the reference category of intake (≤3 times per month or never). Importantly, participants consuming 1 bowl of high-fiber breakfast cereal at least once per week were less likely to have EBP.

To our knowledge, this is the first study focusing on the association of bread consumption with blood pressure status, but also the first including Mexican cereal products such as bolillo, telera, and tortilla. Bread and cereal products are widely consumed by the Mexican population; the 2016 National Health and Nutrition Survey [30] reported that the proportion of subjects consuming sweet cereal (breakfast cereal with sugar or chocolate added, biscuits, sweet bakery goods), was 45.6% at the national level and 54.2% among those living in Mexico City. Mendoza et al. [31] analyzed the cost per calorie of 153 different food items and food groups whose intake was evaluated in the 2012 National Health and Nutrition Survey of Mexico, finding that corn and wheat flour tortillas, tamales, cereals, and grain-based desert were among the foods with the lowest cost per calorie [31]. Additionally, energy intake from wheat products has increased in Mexico during recent decades; Moreno-Altamirano et al. [32] analyzed changes in dietary patterns in the Mexican population and found an increase in the calories provided by wheat products from 246 kcal/person/day in 1961 to 280 kcal/person/day in 2009. A 7-y follow-up cohort study in an urban Mexican population found that a refined-food dietary pattern, characterized by a high consumption of refined grains, corn tortilla, alcohol, and soft drinks was linked to a higher 10-y predicted CVD risk according to the Framingham risk score [33]. 

In our study, after adjustment for age, sex, education level, BMI, alcohol consumption, tobacco use, intake of energy (kcal/day), potassium (mg/day), and magnesium (mg/day), as well as frequency of intake of bacon, ham, sausages and cheeses, consumption of 1 piece of refined-grain products such as bolillo or telera ≥once per week was associated with a significant increased risk of EBP. Even though we did not find a clear adjusted linear association between categories of frequency of intake of bolillo or telera with daily sodium intake, the category representing the highest frequency of intake (≥ once per day) did show a meaningful positive β for daily sodium intake (Table 3). It is important to point out that corn tortilla, a typically low-sodium food (11.4 mg/sodium/1 piece and 40 mg/sodium/100 g), was not associated with daily sodium intake or EBP in this study population. Corn tortilla is a staple food in Mexico and it is present in almost every meal; however, it has been reported that the kcal/day/person from corn-based products in Mexico has decreased in the last decades, in contrast to the increase observed in this same indicator for wheat-based products as highlighted above [32]. 

Results of our study are in agreement with those observed in other populations. Esmaillzadeh et al. [34] showed that adults from Iran consuming ≥281 g/day of refined cereals (white bread [Lavash and Baguette], iceberg bread, noddle, pasta, rice, toasted bread, milled barley, sweet bread, white flour, starch and biscuits) had higher chance of having hypertension (OR: 1.69; 95% CI: 1.10–2.47; *p* for trend 0.04), compared to those consuming <125 g/day of refined cereals. Additionally, in a cross-sectional study in adult urban dwellers from India [35], it was found that the adjusted mean SBP (% difference between Q4 vs. Q1: 2.9; *p* trend = 0.001) and DBP (% difference between Q4 vs. Q1: 1.7; *p* trend = 0.029) increased as quartiles of intake of refined cereals (polish white rice, vermicelli, semolina, refined breakfast cereal, cookies, biscuits, white bread and pastry) increased.

It is also worth noting that the intake of 1 bowl of high-fiber breakfast cereal was linked to a decreased risk of having EBP; however, consumption of packaged whole-grain bread, which is also considered as a high-fiber food, did not exhibit the same relationship with EBP, probably due to its strong and positive adjusted linear association with daily sodium intake, as shown in Table 3. Nonetheless, despite being highly correlated with daily sodium intake, it was not associated with a greater risk of EBP either. Whether fiber content is a factor contributing to mitigate the adverse effects of dietary sodium on blood pressure needs to be further investigated.

In this study population, among those who provided a complete 24 h urinary sample, estimated daily sodium intake, as determined by 24 h urinary sodium excretion, was 3116.3 mg/day (3674.4 in men and 2831.2 in women). These data are in accordance with previous reports of daily sodium intake in two Mexican populations estimated by the same method, where overall mean sodium intake was reported to be 3150 mg/day [7] and 3497.2 mg/day [8]. Since high dietary salt consumption is estimated to cause millions of deaths annually around the world related to cardiovascular events, the Pan-American Health Organization (PAHO) has strongly recommended the implementation of programs and strategies to reduce dietary sodium content in food [36], especially in bread, bakery and other cereal products [14,16]. In the Americas, countries such as Argentina, Brazil, Canada, Chile, Ecuador, Mexico, Paraguay, and the United States have set voluntary or regulatory targets and timelines to reduce salt content in processed food, including bread products [21]. Monitoring of these initiatives in Argentina and Chile has revealed promising data. Argentina reported an average 25% reduction in the sodium content in bread (the authors did not specify what type of bread) from 2011 to 2013, and Chile observed an average decrease in sodium levels in bread from >830 mg/100 g to 479 mg/100 g [37]. In Mexico, a voluntary reformulation initiative at the national level to reduce sodium levels in sliced bread and bolillo, which were estimated to be 520 mg/100 g, was established in 2012 [15]; however, at present, there are no monitoring reports on the progress of this initiative. The results of our study support the need for a continue effort to properly monitoring this initiative. 

Bolillo or telera bread may be considered as a moderate-sodium containing food (approx. 520 mg/100 g); however, the association observed in this present study between the consumption of these food products ≥once per week and EBP may be linked not only to their sodium content, but also to a higher frequency of intake among those individuals classified within this category of intake (≥once per week), which may contribute to a higher daily sodium intake attributed to these food products, and thus leading to a greater related risk of EBP. Further studies in larger populations aimed to evaluate the linear rather than binomial associations between the frequency of intake of bread (packaged sliced bread and bolillo or telera) and blood pressure are still needed.

There are limitations that should be considered when examining the results of this study. Firstly, the cross-sectional design of the study does not make it possible to establish a causal relationship between bread and other cereal products consumption and EBP; however, the FFQ employed to collect dietary data makes it possible to evaluate a long-term exposure (1 year) to the consumption of the studied food items. Secondly, it was not possible to differentiate between certain type of breads and cereals by using this FFQ; for example, rye bread was evaluated within the “packaged sliced whole-grain bread” category, while the “oats” food category included both natural and flavored oats, which are different in sodium content, among other nutrients. Thirdly, the FFQ does not include hot dog and hamburger buns among the evaluated food items. Since the consumption by the urban Mexican population of these packaged products is high [8], these types of cereal products need to be considered in further studies. Fourthly, this studied population is mainly comprised of healthy volunteers who enrolled in the Tlalpan 2020 cohort study and those excluded from this cohort due to hypertension; thus, our results may be not extrapolated to the general population. Finally, the number of subjects within the reference category of intake (≤3 times per month or never) of corn tortilla was too small to be a proper reference group. Larger prospective studies testing the linear association between blood pressure and the consumption of these food products and their interactions are needed. 

## 5. Conclusions

Results of this study showed that consumption of 1 piece of bolillo or telera ≥once per week was associated with an increased risk of EBP in models adjusted for covariates that could affect these relationships. Consumption of 1 bowl of high-fiber breakfast cereal ≥once per week was associated with a decreased risk of EBP. Whether the high content of fiber in these cereals is the main factor contributing to this observed protective effect needs to be further elucidated; moreover, it would also be relevant to compare their effects against other naturally low in sodium whole-grain cereals commonly used as cereal breakfast in our population, such as natural oats (not flavored from packaged) and amaranth. Finally, initiatives to reduce sodium levels in bread products that are largely consumed in the Mexican population are needed to help manage the risk of EBP at the population level. 

## Figures and Tables

**Table 1:** General characteristics of the study sample by sex ^1^.

	Total	Men	Women	*p* Value ^2^
Participants (n)	2011	737	1274	
Age (year)	37.3 ± 9.1	36.5 ± 9.1	37.7 ± 9.0	0.0036
Education level (%)				
<High school	16.2	16.3	16.2	0.284
High school	36.6	34.4	37.8	
≥ Post-secondary	47.2	49.3	46.0	
Current alcohol intake (%)	69.3	77.6	64.5	<0.0001
Current tobacco use (%)	39.9	49.6	34.4	<0.0001
BMI (%)				
Low weight (BMI < 18.5 kg/m^2^)	1.1	0.5	1.5	0.02
Normal weight (BMI: 18.5–24.9 kg/m^2^)	33.4	29.2	35.9	
Overweight (BMI: 25–29.9 kg/m^2^)	40.6	43.0	39.2	
Obesity (BMI: ≥ 30 kg/m^2^)	24.8	27.3	23.4	
Waist circumference (cm)	90.5 ± 12.8	94.8 ± 12.8	88.1 ± 12.1	<0.0001
Elevated blood pressure (SBP ≥ 120 mmHg and/or DBP ≥ 80 mmHg) (%)	26.7	40.4	18.8	<0.001
Systolic blood pressure (mm Hg)	107.8 ± 12.4	112.0 ± 12.3	105.1 ± 11.6	<0.0001
SBP ≥ 120 mm Hg (%)	15.5	25.1	10.0	<0.0001
Diastolic blood pressure (mmHg)	72.7 ± 9.4	75.8 ± 9.3	70.8 ± 8.9	<0.0001
DBP ≥ 80 mm Hg (%)	22.9	35.1	15.9	<0.0001
Fasting plasma glucose (mmol/L)	5.1 ± 0.5	5.2 ± 0.5	5.1 ± 0.5	<0.0001
triglycerides (mmol/L)	1.7 ± 1.1	2.0 ± 1.5	1.5 ± 0.8	<0.0001
Total cholesterol (mmol/L)	4.8 ± 0.9	4.9 ± 1.0	4.8 ± 0.9	0.0084
HDL-c (mmol/L)	1.3 ± 0.3	1.1 ± 0.3	1.3 ± 0.3	<0.0001
LDL-c (mmol/L)	3.1 ± 0.8	3.3 ± 0.8	3.1 ± 0.8	<0.0001

Abbreviations: BMI, body mass index; SBP, systolic blood pressure; DBP, diastolic blood pressure; HDL, high density lipoprotein; LDL, low density lipoprotein. ^1^ Data are presented as mean ± standard deviation for continuous variables and as percentages for categorical variables; ^2^ *p* value for comparison between men and women by independent sample t-test for continuous variables and chi-square test for categorical variables.

**Table 2:** Dietary characteristics of the study sample by sex ^1^.

	Total	Men	Women	*p* Value ^2^
Participants (n)	2011	737	1274	
Energy intake (kcal/day)	2271.5 ± 747.3	2535.7 ± 864.3	2119.2 ± 621.7	<0.0001
Protein (g/day)	81.2 ± 23.3	89.7 ± 34.2	76.3 ± 23.5	<0.0001
Total fat (g/day)	89.7 ± 33.1	97.5 ± 37.7	85.1 ± 28.2	<0.0001
Carbohydrates (g/day)	282.4 ± 96.5	308.9 ± 108.3	267.1 ± 85.2	<0.0001
Potassium intake (mg/day)	3073.4 ± 1051.9	3210.5 ± 1191.4	2994.09 ± 953.6	<0.0001
Magnesium intake (mg/day)	338.62 ± 108.5	366.12 ± 125.5	322.7 ± 93.8	<0.0001
Sodium intake (mg/day)	1806.2 ± 680.2	1968.8 ± 812.3	1712.1 ± 569.9	<0.0001
Urinary sodium excretion (mg/24 h) ^3^	3116.3 ± 1273.3	3674.4 ± 1420.2	2831.2 ± 1086.3	<0.0001
**Frequency of intake of bacon (%)**				
Category 1 (≤3 times per month or never)	92.3	90.0	93.6	0.011
Category 2 (1–4 times per week)	7.4	9.4	6.2	
Category 3 (5–6 times per week)	0.3	0.5	0.1	
Category 4 (≥ once per day)	0.1	0.1	0.1	
**Frequency of intake of sausages (%)**				
Category 1 (≤3 times per month or never)	66.1	59.8	69.8	<0.001
Category 2 (1–4 times per week)	32.1	37.2	29.2	
Category 3 (5–6 times per week)	1.1	1.8	0.7	
Category 4 (≥ once per day)	0.7	1.2	0.3	
**Frequency of intake of ham (%)**				
Category 1 (≤3 times per month or never)	17.5	14.7	19.2	<0.001
Category 2 (1–4 times per week)	65.5	63.5	66.6	
Category 3 (5–6 times per week)	9.4	12.4	7.7	
Category 4 (≥ once per day)	7.6	9.5	6.5	
**Frequency of intake of Oaxaca cheese (%)**				
Category 1 (≤3 times per month or never)	28.3	27.0	29.0	0.601
Category 2 (1–4 times per week)	62.9	63.6	62.4	
Category 3 (5–6 times per week)	4.3	4.2	4.4	
Category 4 (≥ once per day)	4.5	5.2	4.2	
**Frequency of intake of Manchego cheese (%)**				
Category 1 (≤3 times per month or never)	63.4	58.5	66.3	0.001
Category 2 (1–4 times per week)	32.2	36.0	29.0	
Category 3 (5–6 times per week)	2.2	2.3	2.2	
Category 4 (≥ once per day)	2.2	3.3	1.6	
**Frequency of intake of fresh cheese (%)**				
Category 1 (≤3 times per month or never)	24.9	27.1	23.6	0.311
Category 2 (1–4 times per week)	62.8	59.6	62.8	
Category 3 (5–6 times per week)	5.5	4.9	5.5	
Category 4 (≥ once per day)	8.2	8.4	8.2	

^1^ Data are presented as mean ± standard deviation for continuous variables and as percentages for categorical variables; ^2^ *p* value for comparison between men and women by independent sample t-test for continuous variables and chi-square test for categorical variables; ^3^ Urinary sodium excretion was determined for 1492 subjects (507 men and 985 women), for whom the 24 h urine sample was considered as complete (standard creatinine excretion rate for sex).

**Table 3:** Mean daily sodium intake (mg/day) across categories of frequency of intake and linear regression analysis for the association between categories of frequency of intake of food products and daily sodium intake (mg/day).

Categories of Frequency of Intake	n	Dietary Sodium Intake (mg/day) ^1^	*p* Value ^2^	Unadjusted β	95% CI		*p* Value	Adjusted ^3^ β	95% CI		*p* Value
**1 piece of bolillo or telera**											
Category 1 (≤3 times per month or never)	566	1541.96 ± 549.59 ^a^	<0.001	1.00				1.00			
Category 2 (1–4 times per week)	1227	1828.34 ± 598.09 ^b^		286.38	223.09, 349.67		<0.001	12.55	−25.16, 50.26		0.514
Category 3 (5–6 times per week)	86	2085.84 ± 565.8 ^c^		543.88	399.71, 688.05		<0.001	82.69	−2.25, 167.62		0.056
Category 4 (≥ once per day)	132	2551.34 ± 1150.1 ^d^		1009.37	888.97, 1129.77		<0.001	251.36	176.24, 326.49		<0.001
**1 slice of packaged white bread**											
Category 1 (≤3 times per month or never)	1160	1692.45 ± 619.27 ^a^	<0.001	1.00				1.00			
Category 2 (1–4 times per week)	643	1869.3 ± 649.19 ^b^		176.85	113.49, 240.21		<0.001	−25.13	−61.38, 11.12		0.174
Category 3 (5–6 times per week)	98	2072.76 ± 703.06 ^c^		380.30	244.74, 515.87		<0.001	−16.20	−93.31, 60.90		0.680
Category 4 (≥ once per day)	110	2399.52 ± 974.83 ^d^		707.07	578.50, 835.63		<0.001	177.322	102.73, 251.92		<0.001
**1 piece of sweet bakery goods**											
Category 1 (≤3 times per month or never)	440	1583.37 ± 545.88 ^a^	<0.001	1.00				1.00			
Category 2 (1–4 times per week)	1163	1793.21 ± 584.31 ^b^		209.83	137.42, 282.24		<0.001	−8.18	−49.46, 33.09		0.697
Category 3 (5–6 times per week)	142	1967.08 ± 690.42 ^c^		383.71	258.85, 508.58		<0.001	−111.32	−182.99, −39.64		0.002
Category 4 (≥ once per day)	266	2145.77 ± 1025.48 ^c^		562.39	461.91, 662.88		<0.001	−81.37	−142.17, −20.58		0.009
**1 slice of packaged whole-grain bread**											
Category 1 (≤3 times per month or never)	853	1701.33 ± 638.99 ^a^	<0.001	1.00				1.00			
Category 2 (1–4 times per week)	834	1780.39 ± 602.48 ^b^		79.05	16.19, 141.91		0.014	71.18	36.28, 106.01		<0.001
Category 3 (5–6 times per week)	153	1942.37 ± 711.06 ^c^		241.04	127.71, 354.37		<0.001	108.30	45.44, 171.16		0.001
Category 4 (≥ once per day)	171	2333.45 ± 915.29 ^d^		632.12	523.96, 740.28		<0.001	328.67	267.47, 389.87		<0.001
**1 pi** **ece of corn tortilla**											
Category 1 (≤3 times per month or never)	27	1396.49 ± 516.77 ^a^	<0.001	1.00				1.00			
Category 2 (1–4 times per week)	216	1530.48 ± 515.96 ^a^		133.98	−133.02, 400.99		0.325	77.86	−70.85, 226.58		0.305
Category 3 (5–6 times per week)	140	1551.44 ± 456.85 ^a^		154.94	−120.00, 429.88		0.269	41.68	−111.46, 194.83		0.594
Category 4 (≥ once per day)	1628	1871.49 ± 701.06 ^b^		474.99	221.18, 728.81		<0.001	29.74	−112.07, 171.54		0.681
**1 piece of wheat flour tortilla**											
Category 1 (≤3 times per month or never)	1106	1660.56 ± 591.66 ^a^	<0.001	1.00				1.00			
Category 2 (1–4 times per week)	748	1924.61 ± 652.67 ^bc^		264.05	203.33, 324.78		<0.001	9.58	−26.40, 45.56		0.602
Category 3 (5–6 times per week)	64	2081.08 ± 531.81 ^c^		420.52	255.60, 585.43		<0.001	−13.53	−108.59, 81.53		0.780
Category 4 (≥ once per day)	93	2396.78 ± 1214.81 ^d^		736.21	597.72, 874.71		<0.001	52.54	−29.17, 134.25		0.207
**1 bowl of oats (plain or flavoured)**											
Category 1 (≤3 times per month or never)	1210	1766.74 ± 639.21 ^a^	<0.001	1.00				1.00			
Category 2 (1–4 times per week)	610	1848.84 ± 696.77 ^a^		82.10	16.19, 148.00		0.015	103.86	67.99, 139.73		<0.001
Category 3 (5–6 times per week)	92	1748.08 ± 520.09 ^a^		−18.66	−162.19, 124.87		0.799	112.88	34.54, 191.22		0.005
Category 4 (≥ once per day)	99	2079.92 ± 1034.31 ^b^		313.18	174.45, 451.91		<0.001	191.82	116.36, 267.28		<0.001
**1 bowl of refined-grain breakfast cereal**											
Category 1 (≤3 times per month or never)	1261	1721.61 ± 684.85 ^a^	0.002	1.00				1.00			
Category 2 (1–4 times per week)	652	1923.54 ± 619.67 ^b^		201.93	138.53, 265.33		<0.001	10.45	−25.17, 46.06		0.565
Category 3 (5–6 times per week)	54	2205.43 ± 799.67 ^c^		483.81	301.16, 666.47		<0.001	86.75	−15.27, 188.77		0.096
Category 4 (≥ once per day)	44	2001.95 ± 788.06 ^c^		280.34	78.76, 481.92		0.006	0.102	−112.62, 112.82		0.999
**1 bowl of high-fibre breakfast cereal**											
Category 1 (≤3 times per month or never)	1455	1766.47 ± 636.49 ^a^	<0.001	1.00				1.00			
Category 2 (1–4 times per week)	470	1891.199 ± 706.75 ^b^		124.73	54.41, 195.04		0.001	41.42	2.83, 80.00		0.035
Category 3 (5–6 times per week)	50	1833.01 ± 572.93 ^ab^		66.54	−124.08, 257.15		0.494	43.20	−61.17, 147.57		0.417
Category 4 (≥ once per day)	36	2265.31 ± 1484.31 ^c^		498.84	275.24, 722.43		<0.001	269.05	146.43, 391.68		<0.001

^1^ Dietary sodium intake as determined by the food frequency questionnaire; ^2^ *p* value for the comparison of dietary sodium intake between categories of food products intake by one-way analysis of variance. Means with a superscript are significantly different from the other means with a different superscript at a significance level of *p* < 0.05; ^3^ Adjusted linear regression model for age; sex; BMI; energy intake (kcal/day), and frequency of consumption of bacon, ham, sausages and cheeses.

**Table 4:** Logistic regression analysis for the association between consumption of bread and other cereal-derived products at least once per week and elevated blood pressure (EBP).

		Univariable Model			Multivariable Model		
Categories of frequency of intake	n	Unadjusted OR	95% CI	*p* Value	Adjusted OR ^1^	95% CI	*p* Value
**1 piece of bolillo or telera**							
≤3 times per month or never	566	1			1		
≥once per week	1445	1.48	1.13–1.94	0.005	1.39	1.01–1.89	0.041
**1 slice of packaged white bread**							
≤3 times per month or never	1160	1			1		
≥once per week	851	1.15	0.91–1.45	0.248	1.03	0.79–1.35	0.806
**1 piece of sweet bakery goods**							
≤3 times per month or never	440	1			1		
≥once per week	1571	1.15	1.12–2.05	0.007	1.33	0.99–1.78	0.054
**1 slice of packaged whole-grain bread**							
≤3 times per month or never	853	1			1		
≥once per week	1158	0.99	0.77–1.26	0.975	0.98	0.74–1.28	0.864
**1 piece of corn tortilla**							
≤3 times per month or never	27	1			1		
≥once per week	1984	1.28	0.51–3.19	0.597	1.49	0.53–4.23	0.451
**1 piece of wheat flour tortilla**							
≤3 times per month or never	1106	1			1		
≥once per week	905	2.19	0.49–9.83	0.305	1.98	0.42–9.48	0.388
**1 bowl of oats (plain or flavoured)**							
≤3 times per month or never	1210	1			1		
≥once per week	801	0.89	0.73–1.10	0.309	1.03	0.81–1.30	0.804
**1 bowl of refined-grain breakfast cereal**							
≤3 times per month or never	1261	1			1		
≥once per week	750	1.36	0.51–3.65	0.545	1.1	0.84–1.44	0.503
**1 bowl of high-fibre breakfast cereal**							
≤3 times per month or never	1455	1			1		
≥once per week	556	0.74	0.59–0.94	0.012	0.73	0.53–0.98	0.012

^1^ Adjusted Odds Ratio for age; sex; education level; BMI; alcohol consumption; tobacco use; energy (kcal/day), potassium (mg/day), and magnesium (mg/day) intake; and frequency of consumption of bacon, ham, sausages and cheeses.

## References

[1] Rahimi K., Emdin C.A., MacMahon S. (2015). The epidemiology of blood pressure and its worldwide management. Circ. Res..

[2] (2017). Global, regional, and national comparative risk assessment of 84 behavioural, environmental and occupational, and metabolic risks or clusters of risks, 1990–2016: A systematic analysis for the Global Burden of Disease Study 2016. Lancet.

[3] Mills K.T., Bundy J.D., Kelly T.N., Reed J.E., Kearney P.M., Reynolds K., Chen J., He J. (2016). Global Disparities of Hypertension Prevalence and Control: A Systematic Analysis of Population-Based Studies From 90 Countries. Circulation.

[4] Campos-Nonato I., Hernandez-Barrera L., Rojas-Martinez R., Pedroza A., Medina-Garcia C., Barquera-Cervera S. (2013). Hypertension: Prevalence, early diagnosis, control and trends in Mexican adults. Salud Publica de Mexico.

[5] Whelton P.K., Appel L.J., Sacco R.L., Anderson C.A., Antman E.M., Campbell N., Dunbar S.B., Frohlich E.D., Hall J.E., Jessup M. (2012). Sodium, blood pressure, and cardiovascular disease: Further evidence supporting the American Heart Association sodium reduction recommendations. Circulation.

[6] Farquhar W.B., Edwards D.G., Jurkovitz C.T., Weintraub W.S. (2015). Dietary sodium and health: More than just blood pressure. J. Am. Coll. Cardiol..

[7] Vallejo M., Colin-Ramirez E., Rivera Mancia S., Cartas Rosado R., Madero M., Infante Vazquez O., Vargas-Barron J. (2017). Assessment of Sodium and Potassium Intake by 24 h Urinary Excretion in a Healthy Mexican Cohort. Arch. Med. Res..

[8] Colin-Ramirez E., Espinosa-Cuevas A., Miranda-Alatriste P.V., Tovar-Villegas V.I., Arcand J., Correa-Rotter R. (2017). Food Sources of Sodium Intake in an Adult Mexican Population: A Sub-Analysis of the SALMEX Study. Nutrients.

[9] Anderson C.A., Appel L.J., Okuda N., Brown I.J., Chan Q., Zhao L., Ueshima H., Kesteloot H., Miura K., Curb J.D. (2010). Dietary sources of sodium in China, Japan, the United Kingdom, and the United States, women and men aged 40 to 59 years: The INTERMAP study. J. Am. Diet. Assoc..

[10] Gaitan Charry D.A., Estrada A., Argenor Lozano G., Manjarres L.M. (2015). Food sources of sodium: Analysis based on a national survey in Colombia. Nutr. Hosp..

[11] Meneton P., Lafay L., Tard A., Dufour A., Ireland J., Menard J., Volatier J.L. (2009). Dietary sources and correlates of sodium and potassium intakes in the French general population. Eur. J. Clin. Nutr..

[12] Fischer P.W., Vigneault M., Huang R., Arvaniti K., Roach P. (2009). Sodium food sources in the Canadian diet. Appl. Physiol. Nutr. Metab..

[13] Guallar-Castillon P., Munoz-Pareja M., Aguilera M.T., Leon-Munoz L.M., Rodriguez-Artalejo F. (2013). Food sources of sodium, saturated fat and added sugar in the Spanish hypertensive and diabetic population. Atherosclerosis.

[14] Belz M.C., Ryan L.A., Arendt E.K. (2012). The impact of salt reduction in bread: A review. Crit. Rev. Food Sci. Nutr..

[15] Secretaria de Gobernación (2012). Diario Oficial De La Federación. ACUERDO por el que se Recomienda la Disminución del uso de sal Común o Cloruro de Sodio en la Elaboración de pan como una Medida de Prevención de Enfermedades Cardiovasculares, y otras Crónico-Degenerativas.

[16] Quilez J., Salas-Salvado J. (2012). Salt in bread in Europe: Potential benefits of reduction. Nutr. Rev..

[17] Coyne K.J., Baldridge A.S., Huffman M.D., Jenner K., Xavier D., Dunford E.K. (2018). Differences in the sodium content of bread products in the USA and UK: Implications for policy. Public Health Nutr..

[18] Serra-Majem L. (2018). Bread and salt: An indissoluble binomial?. Nutr. Hosp..

[19] Chen J., Huang Q., Shi W., Yang L., Chen J., Lan Q. (2016). Meta-Analysis of the Association Between Whole and Refined Grain Consumption and Stroke Risk Based on Prospective Cohort Studies. Asia-Pac. J. Public Health.

[20] Williams P.G. (2012). Evaluation of the evidence between consumption of refined grains and health outcomes. Nutr. Rev..

[21] Campbell N., Legowski B., Legetic B., Ferrante D., Nilson E., Campbell C., L’Abbe M. (2014). Targets and timelines for reducing salt in processed food in the Americas. J. Clin. Hypertens..

[22] Colin-Ramirez E., Rivera-Mancia S., Infante-Vazquez O., Cartas-Rosado R., Vargas-Barron J., Madero M., Vallejo M. (2017). Protocol for a prospective longitudinal study of risk factors for hypertension incidence in a Mexico City population: The Tlalpan 2020 cohort. BMJ Open.

[23] Whelton P.K., Carey R.M., Aronow W.S., Casey D.E., Collins K.J., Dennison Himmelfarb C., DePalma S.M., Gidding S., Jamerson K.A., Jones D.W. (2017). 2017 ACC/AHA/AAPA/ABC/ACPM/AGS/APhA/ASH/ASPC/NMA/PCNA Guideline for the Prevention, Detection, Evaluation, and Management of High Blood Pressure in Adults: Executive Summary: A Report of the American College of Cardiology/American Heart Association Task Force on Clinical Practice Guidelines. Hypertension.

[24] The International Society for the Advancement of Kinanthropometry (ISAK) (2001). International Standards for Anthropometric Assessment.

[25] Stewart A.D., Marfell-Jones M.J., Olds T., De Ridder J.H. (2011). International Standards for Anthropometric Assessment.

[26] Hernandez-Avila M., Romieu I., Parra S., Hernandez-Avila J., Madrigal H., Willett W. (1998). Validity and reproducibility of a food frequency questionnaire to assess dietary intake of women living in Mexico City. Salud Publica de Mexico.

[27] Hernández-Avila M., Resoles M., Parra S., Romieu I. (2003). SNUT Sistema de Evaluacion de Hábitos Nutricioanles y Consumo de Nutrimentos.

[28] Wielgosz A., Robinson C., Mao Y., Jiang Y., Campbell N.R., Muthuri S., Morrison H. (2016). The Impact of Using Different Methods to Assess Completeness of 24-h Urine Collection on Estimating Dietary Sodium. J. Clin. Hypertens..

[29] Odani S., Armour B.S., Graffunder C.M., Willis G., Hartman A.M., Agaku I.T. (2018). State-Specific Prevalence of Tobacco Product Use Among Adults—United States, 2014–2015. MMWR Morb. Mortal. Wkly. Rep..

[30] Instituto Nacional de Salud Pública (2017). Enuesta Nacional de Salud y Nutrición 2016 Medio Camino.

[31] Mendoza A., Perez A.E., Aggarwal A., Drewnowski A. (2017). Energy density of foods and diets in Mexico and their monetary cost by socioeconomic strata: Analyses of ENSANUT data 2012. J. Epidemiol. Community Health.

[32] Moreno-Altamirano L., Hernandez-Montoya D., Silberman M., Capraro S., Garcia-Garcia J.J., Soto-Estrada G., Sandoval-Bosh E. (2014). The nutrition transition and the double burden of malnutrition: Changes in dietary patterns 1961–2009 in the Mexican socioeconomic context. Arch. Latinoam. Nutr..

[33] Denova-Gutierrez E., Tucker K.L., Flores M., Barquera S., Salmeron J. (2016). Dietary Patterns Are Associated with Predicted Cardiovascular Disease Risk in an Urban Mexican Adult Population. J. Nutr..

[34] Esmaillzadeh A., Mirmiran P., Azizi F. (2005). Whole-grain consumption and the metabolic syndrome: A favorable association in Tehranian adults. Eur. J. Clin. Nutr..

[35] Radhika G., Van Dam R.M., Sudha V., Ganesan A., Mohan V. (2009). Refined grain consumption and the metabolic syndrome in urban Asian Indians (Chennai Urban Rural Epidemiology Study 57). Metab. Clin. Exp..

[36] Legetic B., Campbell N. (2011). Reducing salt intake in the Americas: Pan American Health Organization actions. J. Health Commun..

[37] Campbell N. (2015). Population Level Dietary Salt Reduction Initiative in the Americas. http://resources.cpha.ca/CPHA/Conf/Data/2015/A15-633e.pdf.

